# Supplementary material for: Silver Nanoparticle–Antibiotic Combinations: A Strategy to Overcome Bacterial Resistance in Escherichia coli, Salmonella Enteritidis and Staphylococcus aureus
Source: Antibiotics (Basel). 2025 Sep 24;14(10):960. doi: 10.3390/antibiotics14100960 (PMC12562104; doi:10.3390/antibiotics14100960)
Supplement: Supplementary file 1 [file antibiotics-14-00960-s001.zip › antibiotics-3875951-supplementary.pdf]

Table S1. Fractional Inhibitory Concentration Index (FICI) of combination 1 (AMP + Bio-AgNP) and type of antibacterial interaction.

	AMP (µg/mL)		Bio-AgNP (µM)			
**Bacterial strain**	**MIC[1]**	**MIC[2]**	**MIC[1]**	**MIC[2]**	**FICI**	**Antibacterial interaction**
E. coli ATCC 25922	8	0.25	62.5	15.6	0.28	Synergism
S. Enteritidis ATCC 13076	8	0.12	125	62.5	0.51	Additive
S. aureus ATCC 25923	0.5	0.12	62.5	15.6	0.48	Synergism
E. coli 5616	128	4	62.5	15.6	0.28	Synergism
S. Typhimurium 685	8	1	125	3.9	0.16	Synergism
S. aureus N315	128	16	250	31.2	0.25	Synergism

[1] Minimum Inhibitory Concentration of the individual antimicrobial agent.
[2] Minimum Inhibitory Concentration of the combined antimicrobial agent.

Table S2. Fractional Inhibitory Concentration Index (FICI) of combination 2 (ENRO + Bio-AgNP) and type of antibacterial interaction.

	ENRO (μg/mL)		Bio-AgNP (μM)			
**Bacterial strain**	**MIC[1]**	**MIC[2]**	**MIC[1]**	**MIC[2]**	**FICI**	**Antibacterial interaction**
E. coli ATCC 25922	0.01	0.0005	62.5	15.6	0.3	Synergism
S. Enteritidis ATCC 13076	0.06	0.003	125	62.5	0.55	Additive
S. aureus ATCC 25923	0.12	0.06	62.5	31.2	1	Additive
E. coli 5616	0.015	0.0002	62.5	31.2	0.51	Additive
S. Typhimurium 685	0.03	0.0018	125	62.5	0.56	Additive
S. aureus N315	0.12	0.0009	250	250	1	Additive

[1] Minimum Inhibitory Concentration of the individual antimicrobial agent.
[2] Minimum Inhibitory Concentration of the combined antimicrobial agent.

Table S3. Percentage of decrease in the concentration of compounds in combination compared to individual compounds.

	C1		C2	
**Bacterial strain**	**AMP (%)**	**Bio-AgNP (%)**	**ENRO (%)**	**Bio-AgNP (%)**
E. coli ATCC 25922	96.88	50	95	50
S. Enteritidis ATCC 13076	98.5	50	95	50
S. aureus ATCC 25923	76	75.04	50	50
E. coli 5616	96.88	75.04	98.66	50

S. Typhimurium 685	87.50	96.88	94	50
S. aureus N315	87.5	87.5	99.25	0

Table S4. The fold increase in MIC of the combined agents after prolonged exposure

**Bacterial Strain**	**Increased MIC of combination**			
	**C1**		**C2**	
	**AMP**	**Bio-AgNP**	**ENRO**	**Bio-AgNP**
E. coli ATCC 25922	4x	-	0.5x	4x
S. Enteritidis ATCC 13076	66.6x	-	20x	-
S. aureus ATCC 25923	-	4x	8x	-

Table S5. Susceptibility profile of *E. coli* ATCC 25922 to antibiotics from different classes.

**Antibiotic**	**Inhibition zone (mm)**	**S/R (mm)**	**I (mm)**
Amoxicillin-clavulanate	30	≥18 / ≤13	14-17
Ampicillin	23	≥17 / ≤13	14-16
Cefazolin	25	≥23 / ≤19	20-22
Cefepime	40	≥25 / ≤18	-
Cefoxitin	30	≥18 / ≤14	15-17
Ceftriaxone	34	≥26 / ≤22	20-22
Ciprofloxacin	40	≥26 / ≤21	22-25
Chloramphenicol	35	≥18 / ≤12	13-17
Enrofloxacin	40	≥23 / ≤16	17-22
Fosfomycin	30	≥16 / ≤12	13-15
Gentamicin	27	≥15 / ≤12	13-14
Imipenem	32	≥23 / ≤19	20-22
Nitrofurantoin	27	≥17 / ≤14	15-16
Sulfamethoxazole/Trimethoprim	33	≥16 / ≤10	11-15
Tetracycline	30	≥15 / ≤11	12-14
Tobramycin	25	≥15 / ≤12	13-14

Table S6. Susceptibility profile of *S.* Enteritidis ATCC 13076 to antibiotics from different classes.

Antibiotic	Inhibition zone (mm)	S/R (mm)	I (mm)
Amoxicillin-clavulanate	35	≥18 / ≤13	14-17
Ampicillin	30	≥17 / ≤13	14-16
Cefazolin	30	≥23 / ≤19	20-22
Cefepime	40	≥25 / ≤18	-
Cefoxitin	30	≥18 / ≤14	15-17
Ceftriaxone	30	≥26 / ≤22	20-22
Ciprofloxacin	40	≥26 / ≤21	22-25
Chloramphenicol	30	≥18 / ≤12	13-17
Enrofloxacin	35	≥23 / ≤16	17-22
Fosfomycin	30	≥16 / ≤12	13-15
Gentamicin	30	≥15 / ≤12	13-14
Imipenem	40	≥23 / ≤19	20-22
Nitrofurantoin	25	≥17 / ≤14	15-16
Sulfamethoxazole/Trimethoprim	40	≥16 / ≤10	11-15
Tetracycline	27	≥15 / ≤11	12-14
Tobramycin	25	≥15 / ≤12	13-14

Table S7. Susceptibility profile of *S. aureus* ATCC 25923 to antibiotics from different classes.

Antibiotics	Inhibition zone (mm)	S/R (mm)	I (mm)
Ampicillin	40	≥18 / ≤18	-
Azithromycin	35	≥18 / ≤13	14-17
Cefoxitin	30	≥22 / ≤21	-
Ciprofloxacin	35	≥21 / ≤15	16-20
Clindamycin	40	≥21 / ≤14	15-20
Chloramphenicol	30	≥18 / ≤12	13-17
Enrofloxacin	35	≥23 / ≤16	17-22
Gentamicin	40	≥15 / ≤12	-
Nitrofurantoin	35	≥17 / ≤14	15-16
Sulfamethoxazole/Trimethoprim	40	≥16 / ≤10	11-15
Tetracycline	35	≥19 / ≤14	15-18
Vancomycin	30	-	-
